# Pulmonary blood volume measured by cardiovascular magnetic resonance: influence of pulmonary transit time methods and left atrial volume

**DOI:** 10.1186/s12968-021-00809-1

**Published:** 2021-10-28

**Authors:** Anders Nelsson, Mikael Kanski, Henrik Engblom, Martin Ugander, Marcus Carlsson, Håkan Arheden

**Affiliations:** 1 Clinical Physiology, Department of Clinical Sciences Lund, Skåne University Hospital, Lund University, Lund, Sweden; 2 Department of Clinical Physiology, Karolinska University Hospital, and Karolinska Institutet, Stockholm, Sweden; 3 Kolling Institute, Royal North Shore Hospital, and Charles Perkins Centre, University of Sydney, Sydney, Australia

**Keywords:** Congestion, Heart failure, First-pass perfusion, Pulmonary blood volume, Pulmonary transit time

## Abstract

**Background:**

Increased pulmonary blood volume (PBV) is a measure of congestion and is associated with an increased risk of cardiovascular events. PBV can be quantified using cardiovascular magnetic resonance (CMR) imaging as the product of cardiac output and pulmonary transit time (PTT), the latter measured from the contrast time-intensity curves in the right and left side of the heart from first-pass perfusion (FPP). Several methods of estimating PTT exist, including pulmonary transit beats (PTB), peak-to-peak, and center of gravity (CoG). The aim of this study was to determine the accuracy and precision for these methods of quantifying the PBV, taking the left atrium volume (LAV) into consideration.

**Methods:**

Fifty-eight participants (64 ± 11 years, 24 women) underwent 1.5 T CMR. PTT was quantified from (1) a basal left ventricular short-axis image (FPP), and (2) the reference method with a separate contrast administration using an image intersecting the pulmonary artery (PA) and the LA (CoG(PA-LA)).

**Results:**

Compared to the reference, PBV for (a) PTB(FPP) was 14 ± 17% larger, (b) peak-peak(FPP) was 17 ± 16% larger, and (c) CoG(FPP) was 18 ± 10% larger. Subtraction of the LAV (available for n = 50) decreased overall differences to − 1 ± 19%, 2 ± 18%, and 3 ± 12% for PTB(FPP), peak-peak(FPP), and CoG(FPP), respectively. Lowest interobserver variability was seen for CoG(FPP) (− 2 ± 7%).

**Conclusions:**

CoG(PA-LA) and FPP methods measured the same PBV only when adjusting for the LAV, since FPP inherently quantifies a volume consisting of PBV + LAV. CoG(FPP) had the best precision and lowest interobserver variability among the FPP methods of measuring PBV.

**Graphical abstract:**

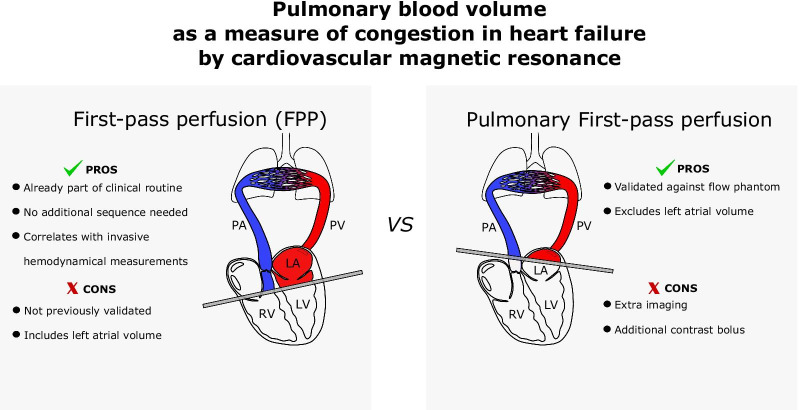

## Background

**Fig. 1:**
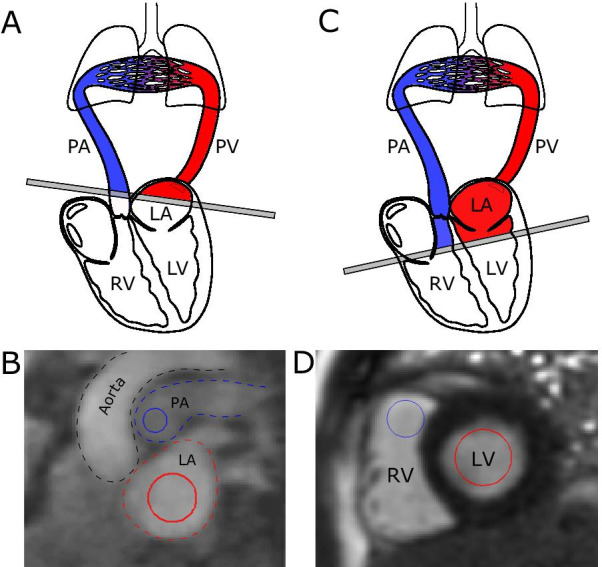
Cardiovascular magnetic resonance (CMR) slices and positioning of the regions of interest. **A** Slice position for pulmonary transit time pulmonary artery-left atrium [CoG(PA-LA)]; **B** corresponding CMR image and the positioning of the regions of interest (ROIs) in the pulmonary artery (PA) in blue and the left atrium (LA) in red. **C** slice position for first-pass perfusion (FPP); **D** corresponding CMR image and the positioning of ROIs in the right ventricular outflow tract (RVOT) in blue and basal left ventricle (LV) in red. *PV* pulmonary veins

Left-sided heart failure leads to congestion through increased filling pressures with distended pulmonary vasculature, which potentially could result in an increased pulmonary blood volume (PBV). Classical invasive measurements of PBV used pulmonary transit time (PTT) and found prolonged PTT in mitral stenosis [1, 2], and correlation of PTT to mean left atrial (LA) pressure [1]. We have previously shown and validated with a flow phantom how PBV can be non-invasively quantified with cardiovascular magnetic resonance (CMR) as the product of cardiac output (ml/s) from phase-contrast flow and PTT (s) from a high temporal resolution first-pass sequence [3]. In that and subsequent studies [4], PTT was measured as the transit time for an intravenous contrast bolus between the main pulmonary artery and the left atrium (PA-LA). In the phantom validation, the center-of-gravity (CoG) approach proved the most precise and reproducible [3]. This method of calculating PBV will in this work be referred to as CoG(PA-LA). The PROVE-HF study, where PBV was measured by CMR from a clinical first-pass perfusion (FPP) sequence, showed that heart failure outpatients who present with an elevated PBV indexed to body surface area (PBVI) had an increased risk of major adverse cardiac events [5]. Also, PBVI has been found to be increased in hypertrophic cardiomyopathy [6] and in adults with congenital heart disease [7]. PTT has also been found to correlate with reduced ejection fraction [8]. These findings may therefore have significant implications in the management of patients with heart failure. Using FPP for quantification of PBV would be a major advantage since FPP is readily available in the clinical setting, while the reference CoG(PA-LA) requires separate imaging and contrast injection. However, the PBV by FPP will inherently be overestimated compared to CoG(PA-LA) since the left atrial volume (LAV) is included in FPP, but not in CoG(PA-LA) (Fig. 1). Furthermore, the use of different methods for measuring PTT using FPP makes inter-study comparison cumbersome. Therefore, the purpose of this study was to determine the accuracy and precision for different methods of quantifying PBV from FPP sequences using the CoG(PA-LA) method as reference standard, taking the LAV into consideration.

## Methods

The regional ethical committee in Lund, Sweden, approved the study (Dnr 2017/829), which complies with the Declaration of Helsinki. All participants provided written informed consent after receiving written and oral information. Fifty-eight participants without severe valvular lesions were included, whereof 21 were healthy controls and 37 were patients referred for a clinical cardiovascular magnetic resonance (CMR). All participants underwent CMR imaging at 1.5 T (MAGNETOM Aera, Siemens Healthineers, Erlangen, Germany). Patients had different protocols depending on referral. 55/58 (95%) patients underwent stress perfusion imaging with adenosine, and the remaining 3 patients did not undergo stress perfusion. All scanning protocols included balanced steady-state free precession (bSSFP) images in the left ventricular (LV) 2-, 3-, 4-chamber and short-axis views, through-plane flow measurement in the pulmonary artery, FPP imaging at rest, and pulmonary transit time between pulmonary artery to left atrium [CoG(PA-LA)]. Retrospective electrocardiographic (ECG) gating was used.

All image analysis was done using the freely available software Segment (version 2.0 R6246, Medviso, Lund, Sweden) [9].

### Cardiac output

Cardiac output (CO) was measured by CMR flow measurement in a cross section of the pulmonary trunk using a through-plane phase-contrast velocity-encoded gradient echo sequence with retrospective ECG triggering during free breathing and the number of signal averages was 1, as previously described [10]. Flow measurement was also validated using a pulsatile flow phantom resulting in − 1 ± 3% and 3 ± 1% (bias ± SD) in transversal and double-oblique slices respectively, after applying linear background correction. Typical scan parameters were: slice thickness 5 mm; frames per cardiac cycle 35; flip angle 20°; pixel size 1.5 × 1.5 mm; velocity encoding 200 cm/s; TR/TE 9.8/2.7 ms.

### Left atrial volume

Cine images acquired in the short-axis plane covering the LA (no slice gap) were obtained in 50 participants. In the remaining 8 participants, no short-axis cine images of the atria were available for quantification of atrial volume. The short-axis images were delineated at atrial end systole and end diastole, and the average value was then calculated to assess the mean LAV (LAV_mean_) [11]. Images were acquired in end-expiratory breath-hold using bSSFP cine sequences with retrospective ECG triggering. Typical scan parameters were: slice thickness 8 mm; acquired spatial resolution 2.0 × 2.0 mm; flip angle 70°; TR/TE 41/1.1 ms.

### Pulmonary transit time and pulmonary blood volume

The PTT was quantified at rest with two different sequences at least 2 min apart: (1) With CoG(PA-LA) as the reference method, and (2) with FPP used for diagnosing myocardial ischemia using a prototype sequence [12]. For both methods, 0.5 mmol/ml gadoteric acid (Dotarem, Gothia Medical, Billdal, Sweden; or Clariscan, GE Health Care, Danderyd, Sweden) was used as contrast agent and injected at 4 ml/s followed by 20 ml saline flush in the same arm.

#### CoG(PA-LA)

During the injection of a 2 ml contrast agent bolus, imaging was performed using a saturation recovery single-shot bSSFP with minimum saturation time sequence in an image plane intersecting the main pulmonary artery (MPA) and LA. Typical scan parameters were: slice thickness 20 mm; spatial resolution 2.7 × 2.7 mm; temporal resolution 100 ms; flip angle 50°; TR/TE 88/1.2 ms [3].

#### First Pass Perfusion

During the injection of a 0.05 mmol/kg body weight contrast agent bolus, a time-resolved FPP image was acquired as a part of a dual-sequence, quantitative myocardial perfusion mapping scan [12]. This image was acquired in a short-axis plane intersecting the basal LV and the right ventricular outflow tract (RVOT). Typical scan parameters were: slice thickness 8 mm; number of slices 1 (the most basal of 3); spacing between slices 21 mm; spatial resolution 1.9 × 1.9 mm; temporal resolution 1 acquisition per heart beat; flip angle 50 degrees; TR/TE 142/1.0 ms.

**Fig. 2:**
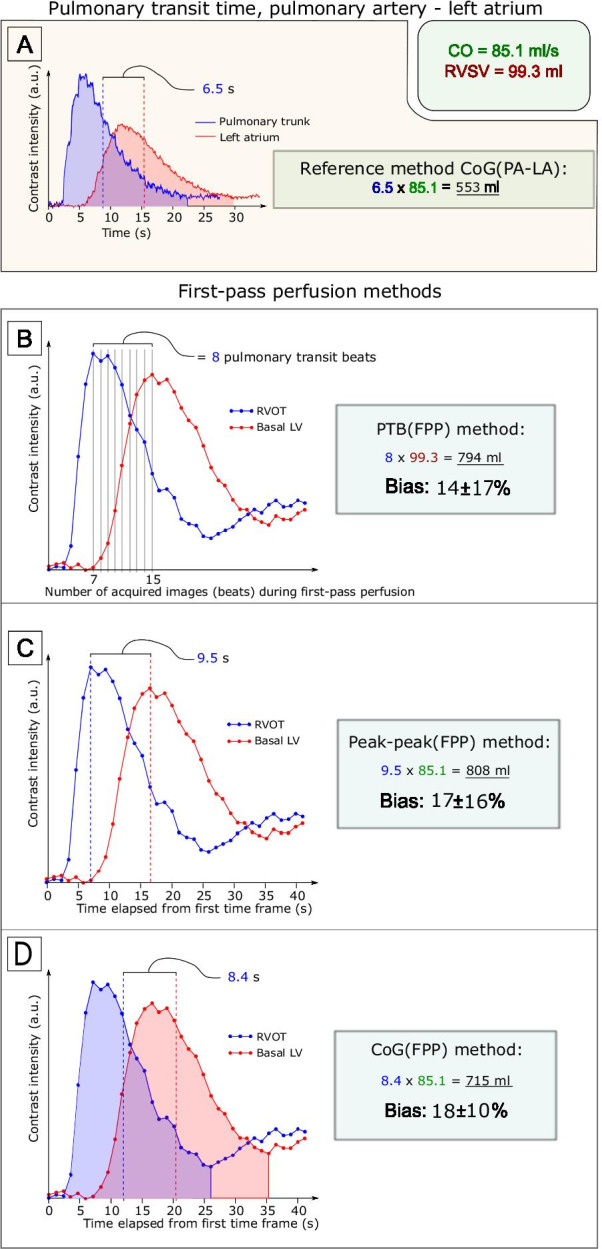
Measurements and pulmonary blood volume (PBV) results from one patient. **A** Measurement of pulmonary transit time (PTT) from the pulmonary transit time pulmonary artery-left atrium (CoG(PA-LA)). **B** First-pass perfusion (FPP) method from the PROVE-HF study [4] using pulmonary transit beats (PTB(FPP)). Panel C shows the FPP peak-peak method (peak-peak(FPP)). **D** FPP center-of-gravity (CoG(FPP)) method. The blue lines indicate contrast intensity measurement in the pulmonary artery (**A**) or basal right ventricle (**B**, **C** and **D**); red lines indicate measurement in the left atrium (**A**) or basal left ventricle (**B**, **C** and **D**). *CO* cardiac output; *RVSV* right ventricular stroke volume

PBV was quantified as the product of CO from flow imaging, and PTT using the information from the bolus injection in four different ways:

1. *Center of Gravity (CoG) between the Pulmonary Artery and Left Atrium [CoG(PA-LA)]* (s) × CO (ml/s) (as previously published by Ugander et al*.* [3]). In short, circular fixed regions-of-interest (ROIs) were manually positioned in the MPA and LA (Fig. 1B). The PTT was defined as the time between the respective curves’ center of gravity (CoG) (Fig. 2A). PBV was calculated as CoG(PA-LA) (s) × CO (ml/s).
2. *Pulmonary transit beats (PTB) [PTB(FPP)],* (as previously published by Ricci et al*.* [5]): ROIs were manually positioned in the most basal of the three FPP image planes acquired. This means that the ROIs were placed in the RVOT and in the basal LV (see Fig. 1D). The number of beats (= PTB) was calculated as the number of images registered between the maximum signal intensity of the ROIs in the RVOT and the LV, respectively. PBV was calculated as the PTB (n) × RVSV (ml) from MPA flow imaging (Fig. 2B).
3. *Peak-peak(FPP)* For each time-intensity curve, the time stamp at peak signal intensity was extracted as described by Ricci et al*.* [6] (Fig. 2C). In contrast to the PROVE-HF method, the time difference between the peaks was used instead of the number of PTB. This means that variation in heart rate during data acquisition was taken into consideration. The peak-peak(FPP) was defined as the time between the respective curves’ peak signal intensities. PBV was calculated as peak-peak(FPP) (s) × CO (ml/s).
4. *CoG(FPP):* For each time-intensity curve, the time point for the CoG was extracted (Fig. 2D). This means that bolus-dilution mechanics were also taken into consideration. The CoG(FPP) was defined as the time between the respective curves’ center of gravity. PBV was calculated as CoG(FPP) (s) × CO (ml/s).

PBV was indexed to body surface area (BSA), calculated using the Mosteller formula. LA volume was corrected for by subtracting LAV_mean_ from PBV calculated with FPP methods.

### Reproducibility

Independent evaluation of PBV by methods 1–4 as described above was performed by two observers with one and 12 years of CMR experience, respectively. All FPP measurements were also performed twice by the same observer.

### Statistics

Data are presented as mean ± standard deviation (SD). Correlation between measurements was calculated using Pearson’s correlation coefficient. Differences between healthy controls and patients were tested with Mann–Whitney U-test. Intraclass correlation (ICC) was performed as a two-way random model with absolute agreement. Bland–Altman plots were generated with the 95% limit of agreement. Differences in SD were tested with the F-test. The F-test and the ICC analyses were performed in SPSS (version 27, Statistical Package for the Social Sciences, International Business Machines, Inc., Armonk, New York, USA). All other statistical analyses were performed in GraphPad Prism 8.3.0 for Windows (GraphPad Software, La Jolla, California, USA). A p-value < 0.05 was considered statistically significant. The following sample size calculation was used: minimum acceptable reliability (ICC) (ρ0): 0.6; expected reliability (ICC) (ρ1): 0.8; significance level (α): 0.05; two-tailed power (1 − β): 80%; number of raters/repetitions per subject (k): 2; expected dropout rate: 10%; sample size; n = 49 with 10% dropout, n including dropout = 55 [13].

## Results

Table 1 shows participants’ characteristics, including patients with different cardiac diseases and patients with normal exams.

**Table 1:** Participant characteristics and cardiovascular magnetic resonance anatomic data

	Healthy controls N = 21	Patients N = 37
Sex (n, female/male)	13/8	11/26
Age (years)	61 ± ﻿8	65 ± 13
BSA (m^2^)	1.9 ± 0.2	2.0 ± 0.2
LV EDV (ml)	147 ± 21	191 ± 49^a^
LV ESV (ml)	56 ± 13	97 ± 44^a^
LV SV (ml)	89 ± 14	94 ± 19^a^
LV EF (%)	61 ± 6	51 ± 11^b^
CO (L/min)	5.5 ± 1.2	5.7 ± 1.4
LA max volume (ml)	92 ± 21	116 ± 29
LA min volume (ml)	43 ± 21	67 ± 27
LAV_mean_ (ml)	68 ± 20	91 ± 27
Etiology (n)	Not applicable	Ischemic heart disease (14), Idiopathic heart failure (8), heart failure with preserved ejection fraction (8), heart transplanted (2), dilated cardiomyopathy (2), myocarditis (1), heart failure due to chemotherapy (1), pericardial cyst (1)

*BSA* body surface area; *LV* left ventricular; *EDV* end-diastolic volume; *ESV* end-systolic volume; *SV* stroke volume; *EF* ejection fraction; *CO* cardiac output; *LA* left atrium; *LAV*_mean_ left atrial mean volume; *CMR* cardiovascular magnetic resonance

^a^Left ventricular volumetry by CMR available in 36 of 37 patients

^b^EF by echocardiography in one patient

### Pulmonary blood volume

**Fig. 3:**
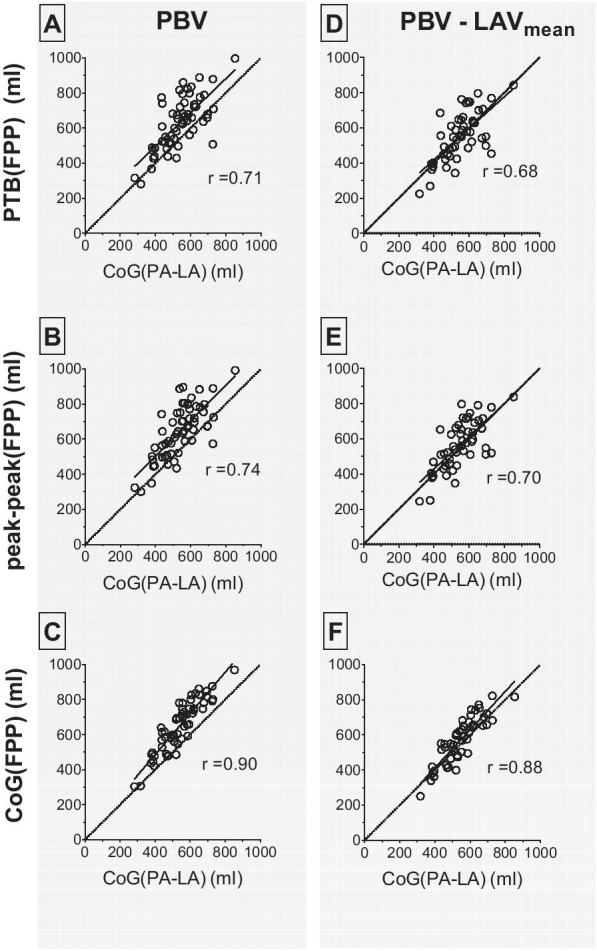
Pulmonary blood volume (PBV). Scatter plots with PBV from pulmonary transit time for pulmonary artery-left atrium (CoG(PA-LA)) as reference compared with **A** pulmonary transit time for pulmonary transit beats (PTB(FPP)), **B** pulmonary transit time for peak to peak (peak-peak(FPP)), and **C** pulmonary transit time for center of gravity (CoG(FPP)). Scatter plots after adjustment for left atrial volumes (LAV) for **D** PTB(FPP), **E** peak-peak(FPP), and **F** CoG(FPP). Dotted lines are the line of identity

**Fig. 4:**
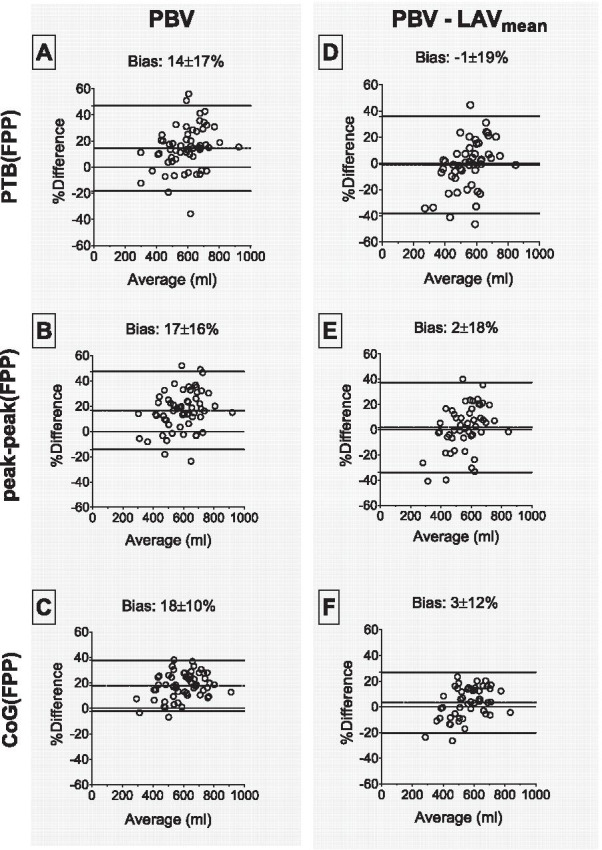
Bland–Altman plots of pulmonary blood volume (PBV). Bland–Altman plots for PBV for pulmonary transit time for pulmonary artery-left atrium (CoG(PA-LA)) compared with **A** pulmonary transit time for pulmonary transit beats (PTB(FPP)), **B** pulmonary transit time for peak to peak (peak-peak(FPP)), and **C** pulmonary transit time for center of gravity (CoG(FPP)). Bland–Altman plots after adjustment for mean left atrial volume (LAV_mean_) for **D** PTB(FPP), **E** peak-peak(FPP), and **F** CoG(FPP). Dotted lines in Bland–Altman plots represent the mean and 95% limits of agreement

Results are described in Tables 2, 3, and 4 and Figs. 3 and 4. Highest precision (lowest variability) was seen comparing CoG(FPP) to CoG(PA-LA) (bias 18 ± 10%, Figs. 3C and 4C). Adjusting for LAV_mean_ (n = 50) resulted in differences in the offset for the FPP methods PTB(FPP), peak-peak(FPP) and CoG(FPP) (from 14 ± 17 to − 1 ± 19%, from 17 ± 16 to 2 ± 18%, and from 18 ± 10 to 3 ± 12%, respectively, Fig. 4D–F). Limits of agreement are presented in Table 3. The F-test showed statistically significant differences in the SDs for all FPP methods compared to CoG(PA-LA) except for PTB(FPP)-LAV_mean_ and peak-peak(FPP)-LAV_mean_.

**Table 2:** Pulmonary blood volume (PBV) in absolute numbers and indexed for body surface area (PBVI) for healthy controls and patients

	PBV (ml) Healthy controls N = 21	PBV (ml) Patients N = 37	PBVI (ml/m^2^) Healthy controls	PBVI (ml/m^2^) Patients	p-values (PBV/PBVI)
CoG(PA-LA)	526 ± 87	549 ± 123	282 ± 43	270 ± 53	0.35/0.18
PTB (FPP)	587 ± 110	656 ± 164	314 ± 57	323 ± 77	0.046*/0.82
PTB (FPP)–LAV_mean_	522 ± 110	572 ± 155	280 ± 58	282 ± 75	0.14/0.87
peak-peak (FPP)	607 ± 112	668 ± 165	324 ± 55	328 ± 76	0.08/0.77
peak-peak (FPP)–LAV_mean_	544 ± 107	589 ± 154	291 ± 54	288 ± 75	0.17/0.84
CoG(FPP)	615 ± 115	672 ± 159	329 ± 56	330 ± 70	0.06/0.96
CoG FPP)–LAV_mean_	551 ± 115	590 ± 143	295 ± 58	291 ± 66	0.18/0.88

*CoG(PA-LA)* pulmonary transit time pulmonary artery – left atrium; *PTB(FPP)* pulmonary transit time, pulmonary transit beats; *peak-peak(FPP)* pulmonary transit time, peak to peak; *CoG(FPP)* pulmonary transit time, center of gravity; *LAV*_*mean*_ mean left atrial volume

Note that patients are unselected, not necessarily patients with heart failure. Asterisk denotes statistically significant difference (p < 0.05)

**Table 3:** Measurements of pulmonary blood volume (PBV) for all participants

	PBV (ml)	PBV, indexed (ml/m^2^)	PBV–LAV_mean_ (ml)	PBV–LAV_mean_, indexed (ml/m^2^)	Pulmonary transit time (s)	Cardiac output (ml/s)	Pulmonary transit beats (n)	RVSV (ml)	F-test PBV vs CoG(PA-LA)	F-test PBV–LAV_mean_ vs CoG(PA-LA)
CoG(PA-LA)	541 ± 111	280 ± 48	–	–	5.7 ± 1.4	93.4 ± 22.5	–	–	–	–
PTB(FPP)	631 ± 150	322 ± 70	552 ± 140	276 ± 78	–	–	8 ± 2	84.5 ± 16.7	< 0.001	0.84
peak-peak(FPP)	644 ± 151	330 ± 70	567 ± 139	283 ± 78	7.1 ± 1.8	93.4 ± 22.5	–	–	< 0.001	0.20
CoG(FPP)	651 ± 145	334 ± 64	573 ± 131	286 ± 73	7.1 ± 1.5	93.4 ± 22.5	–	–	< 0.001	0.01

Results for different methods of measuring PBV, including adjustment of PBV for mean left atrial volume (LAV_mean_). Indexation for PBV by body surface area (BSA). Note that PBV for CoG(PA-LA) excludes the LAV, see Fig. 1

*PTB(FPP)* pulmonary transit time, pulmonary transit beats; *peak-peak(FPP)* pulmonary transit time, peak to peak; *CoG(FPP)* pulmonary transit time, center of gravity; *RVSV* right ventricular stroke volume

**Table 4:** Statistical analysis

		Pearson r	Equation	Bias	95% limits of agreement	p-value	Interobserver bias (%)
PBV	PTB(FPP)	0.71	y = 0.96x + 110	14 ± 17%	− 18 to 47%	< 0.001	− 2 ± 11
	Peak-peak(FPP)	0.74	y = 1.01x + 98	17 ± 16%	− 14 to 47%	< 0.001	0 ± 10
	CoG(FPP)	0.90	y = 1.18x + 14	18 ± 10%	− 2 to 38%	< 0.001	− 2 ± 7
PBV–LAV_mean_	PTB(FPP)	0.68	y = 0.90x + 60	− 1 ± 19%	− 38 to 36%	< 0.001	
	Peak-peak(FPP)	0.70	y = 0.93x + 59	2 ± 18%	− 34 to 37%	< 0.001	
	CoG(FPP)	0.88	y = 1.09x− 23	3 ± 12%	− 20 to 27%	< 0.001	

Descriptive statistics of pulmonary blood volume (PBV) calculated by the different first pass perfusion (FPP) methods compared with pulmonary transit time pulmonary artery – left atrium (CoG(PA-LA))

*PTB(FPP)* pulmonary transit time, pulmonary transit beats; *peak-peak(FPP)* pulmonary transit time, peak to peak; *CoG(FPP)* pulmonary transit time, center of gravity; *LAV*_*mean*_ mean left atrial volume

### Interobserver and intraobserver variability

Results are shown in Tables 3, 4, 5, 6 and 7. Lowest interobserver variability with Bland–Altman was seen for calculation of PBV using the CoG(FPP) method (− 2 ± 7%). PTB(FPP) and peak-peak(FPP) showed similar interobserver variability of − 2 ± 12% and 0 ± 10%, respectively.

**Table 5:** Intraclass correlation

		ICC single measure	Single measure 95% CI	ICC average measure	Average measure 95% CI
PBV	PBT(FPP)	0.56	0.01 to 0.78	0.71	0.18 to 0.87
	Peak-peak(FPP)	0.55	0.01 to 0.79	0.71	0.01 to 0.88
	CoG(FPP)	0.64	− 0.08 to 0.88	0.78	− 0.18 to 0.94
PBV–LAV_mean_	PBT(FPP)	0.66	0.47 to 0.79	0.80	0.64 to 0.88
	Peak-peak(FPP)	0.68	0.49 to 0.80	0.81	0.66 to 0.89
	CoG(FPP)	0.84	0.71 to 0.91	0.91	0.83 to 0.95

Intraclass correlation (ICC) of pulmonary blood volume (PBV) for the different first pass perfusion (FPP) methods compared with pulmonary transit time pulmonary artery-left atrium (CoG(PA-LA))

*PTB(FPP)* pulmonary transit time, pulmonary transit beats; *peak-peak(FPP)* pulmonary transit time, peak to peak; *CoG(FPP)* pulmonary transit time, center of gravity; *LAV*_mean_ mean left atrial volume, *CI* confidence interval

p-values for all correlations were < 0.001

**Table 6:** Interobserver intraclass correlation

		ICC single measure	Single measure 95% CI	ICC average measure	Average measure 95% CI
PBV	PBT(FPP)	0.88	0.80–0.92	0.93	0.89–0.96
	Peak-peak(FPP)	0.92	0.87–0.95	0.96	0.93–0.98
	CoG(FPP)	0.97	0.94–0.98	0.98	0.97–0.99
PBV–LAV_mean_	PBT(FPP)	0.84	0.74–0.91	0.91	0.85–0.95
	Peak-peak(FPP)	0.89	0.82–0.94	0.94	0.90–0.97
	CoG(FPP)	0.95	0.92–0.97	0.98	0.96–0.99

Intraclass correlation (ICC) of pulmonary blood volume (PBV) for intraobserver analysis

*FPP* first pass perfusion; *PTB(FPP)*  pulmonary transit time, pulmonary transit beats; *peak-peak(FPP)*  pulmonary transit time, peak to peak; *CoG(FPP)*  pulmonary transit time, center of gravity; *LAV*_*mean*_ mean left atrial volume, *CI* confidence interval

p-values for all correlations were < 0.001

**Table 7:** Intraobserver intraclass correlation

		ICC single measure	Single measure 95% CI	ICC average measure	Average measure 95% CI
PBV	PBT(FPP)	0.85	0.76–0.91	0.92	0.86–0.95
	Peak-peak(FPP)	0.86	0.77–0.91	0.92	0.87–0.95
	CoG(FPP)	0.97	0.95–0.98	0.99	0.98–0.99

Intraclass correlation (ICC) of pulmonary blood volume (PBV) for intraobserver analysis

*FPP* first pass perfusion; *PTB(FPP)*  pulmonary transit time, pulmonary transit beats; *peak-peak(FPP)*  pulmonary transit time, peak to peak; *CoG(FPP)*  pulmonary transit time, center of gravity; *LAV*_*mean*_ mean left atrial volume, *CI* confidence interval

p-values for all correlations were < 0.001

## Discussion

This study is a head-to-head comparison of three different methods for quantifying the PBV using a FPP sequence, with the validated CoG(PA-LA) as the reference method. It showed that (1) quantification of PBV using FPP images yields about 15–20% higher values compared to the reference method CoG(PA-LA), mainly explained by the inclusion of LAV in the FPP approach since images are acquired in the LV instead of the LA, and (2) the CoG approach is the most robust method with regard to interobserver variability. Thus, using a CoG approach may lead to an increased precision of PBV measurements compared to using the peak-to-peak method or the number of PTB as in PROVE-HF.

### Clinical relevance

Congestion in heart failure is an important prognostic factor in acute [14] and chronic heart failure [15, 16]. The degree of congestion is difficult to quantify, but proxy measures have been used to assess congestion by invasive procedures such as right-heart catheterization, chest X-ray, or clinical signs such as rales on lung auscultation or ankle edema. Congestion is defined as elevated LV diastolic pressure [17], and acute forward failure due to decreased contractility or increased afterload can lead to redistribution of fluid into the pulmonary circulation [18]. A measure of PBV could therefore represent an objective and quantitative measure of congestion. The PROVE-HF study showed worse prognosis for heart failure outpatients with an increased PBVI measured by CMR [5]. The clinical relevance of these data pertains to the possibility of quantifying PBV as a measure of congestion without any additional sequences in a routine clinical CMR perfusion examination using FPP for assessment of myocardial perfusion.

### Relation to earlier studies

Several different methods have previously been used to calculate the PBV with CMR in patients [4–8] and there is no consensus on which method to use, making inter-study comparison difficult. To our knowledge, however, the only method that has been validated is the CoG(PA-LA) method [3], and this method was therefore used as the reference method in this study.

The present study describes how measurements of PBV with FPP are achieved with the highest accuracy and precision using CoG(PA-LA) as reference. Since the CoG approach used in the reference method has shown a smaller offset compared to peak to peak (4 ± 3% vs 10 ± 2%) in a flow phantom setting [3], adapting the CoG approach to the FPP method could potentially increase the accuracy.

### Differences in PBV between FPP and CoG(PA-LA)

Calculation of a volume (V) can be done by multiplying flow (Q) and transit time (t) giving the formula V = Q × t, which is valid for a system with a single input and a single output even with parallel pathways inside [19]. Since the flow (Q) in the MPA can be readily measured by CMR [20], and the anatomical entry and exit boundaries for the flow are determined by the respective region of interest (ROI), the main challenges in calculating PBV are to define the transit time (t) and to choose the entry and exit boundaries.

**Fig. 5:**
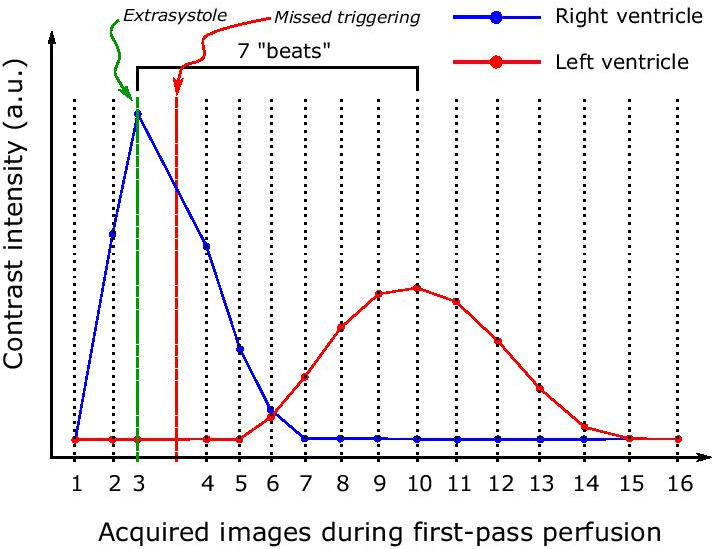
Schematic figure of contrast-intensity measurement using first-pass perfusion imaging with extrasystole and missed triggering. Contrast intensity is shown on the y-axis, and number of acquired images during first-pass perfusion on the x-axis. The blue line denotes the contrast intensity in the right ventricle; red line denotes left ventricle. The green vertical line indicates an extrasystole, and the red vertical line indicates a missed triggering. Note that the number of heart beats is affected by a missed triggering (x-axis), leading to a false number of PTBs and subsequent underestimation of the pulmonary blood volume using the PTT from pulmonary transit beats. Methods using time on the x-axis and center of gravity approaches would be less affected

Using images acquired with FPP, the PROVE-HF approach utilizes the number of heart beats (PTB) between the peaks of the time-intensity curves for each ROI [5–7]. PTB is multiplied by the RV stroke volume, yielding PBV using the PTB(FPP) method. Caveats are that arrhythmias or skipped beats due to missed triggering could affect the number of PTB (see Fig. 5) and that RV stroke volume is not the net volume to the pulmonary circulation as it includes regurgitant volumes of the tricuspid and pulmonary valves. Interestingly, the number of PTBs alone has been shown to correlate with several invasive hemodynamic parameters, including pulmonary capillary wedge pressure and LV end-diastolic pressure [8]. The peak-peak(FPP) method [6] measures t as the time difference between the peak values for the time-intensity curves in each ROI, which means heart rate variation is accounted for and it is less prone to miscalculations due to missed triggering. In the present study, the peak-peak(FPP) method performed similarly to the PTB(FPP) when compared to the CoG(PA-LA) method (14 ± 17% vs 17 ± 16%), which is in line with the findings by Ricci et al.[6].

An alternative to the use of peak-to-peak methods is to calculate the time difference between the centroids of the respective contrast bolus arrivals, which is mathematically more correct, as previously described [19] (see Fig. 2). The CoG is defined as the time point where half of the total signal intensity has reached the ROI. The peak signal intensity can be more affected by placement of the ROI since an ROI can yield two peaks with similar signal intensity but separated in time by 2–3 heart beats (see Fig. 2, panel C for an example). This can make it difficult to know which peak value represents the true peak signal intensity and thus possibly greatly affect t, while t from the CoG method would not be affected. This is in line with the current study that shows higher precision and a lower interobserver variability for CoG(FPP) than the PROVE-HF and peak-to-peak methods.

PBV with CoG(PA-LA) and FPP use different imaging planes to measure the passage of a contrast bolus through the pulmonary circulation (Fig. 1). PBV with CoG(PA-LA) measures the blood volume between the PA-LA (Fig. 1, panels A and B). By comparison, PBV with FPP measures the blood volume between the RV and the LV, and thus includes the LA volume (Fig. 1C and D). While LAV measured with the biplane method was not found to correlate with PTT by Cao et al*.* [8], the prognostic value of PBV from FPP techniques might be affected by different LA sizes. Several studies have shown the relationship between enlarged LA and, for example, atrial fibrillation [21], increased end-diastolic filling pressures [22], and increased all-cause mortality for patients referred for a clinical CMR [23]. Measurements with CoG(PA-LA) results in what can be called the PBV whereas measurements with FPP methods results in what may be called PBV + LAV. We suggest that PBV assessed with FPP should be referred to as PBV + LAV since it cannot be used interchangeably with PBV assessed with CoG(PA-LA) and it should be clear to the clinician which volume has been quantified.

Both PBV and PBV + LAV are likely to carry prognostic information. Increased PBV and increased LAV are both measures of congestion and increased PBV + LAV is likely to also be a measure of congestion. Increased PBV + LAV could be due to increase PBV or increased LAV, or an increase in both. Notwithstanding, PBV + LAV is the easier method to obtain in clinical routine and more likely to be used on a routine basis. We quantified the LAV using the gold standard cine short-axis images, but LAV can also be quantified from biplane area-length or transversal slices [24] which is more available in the clinical routine.

### Indexing of PBV

In this study, we indexed the PBV values to the BSA. While BSA is easy to calculate and has been used in several studies investigating PBV [5–7], it is not necessarily the best anthropometric variable to index against. The BSA does not necessarily reflect the pulmonary vascular system, especially when extrapolating the Mosteller formula in extreme cases of obesity, cachexia, or bodybuilding. A more physiologically appealing entity to consider would be the volume of the lungs. This, however, requires extra sequences and extra measurements for indexing. Also, this approach would have caveats such as patients with emphysema who have larger lungs with large air pockets (bullae) and relatively less lung parenchyma. Thus, there appears to be no perfect metric to index the pulmonary circulation, and BSA or body height appear to be the most practical way for indexation in clinical routine.

### Limitations

Our study has several limitations. One limitation is that CoG(FPP) uses the same calculation method as CoG(PA-LA) and therefore has an inherent advantage over the other methods. The validation of the CoG(PA-LA) method, however, showed that CoG was the best method using a phantom [3], and therefore is a justifiable reference method. The measurements should ideally be evaluated in light of a true standard reference measurement of PBV. Such a measurement, however, does not exist and we believe that the CoG(PA-LA) method is the closest to a reference method. In FPP, the dynamics of signal intensity in the blood pool during the contrast bolus injection is not linear in relation to the changes in gadolinium concentration. However, previous data from phantom validation have shown excellent agreement between PBV from direct measurements (timer and beaker) and calculation from CoG(PA-LA) [3]. A large contrast bolus, meaning a high gadolinium concentration, could increase the non-linearity of the signal intensity. A high enough concentration could also lead to signal intensity saturation which would affect the absolute peaks. The same effects could happen with a very fast injection of contrast. A too slow injection speed would yield flatter but more extended curves, where recirculation of contrast could affect the curves. The first contrast bolus injection for CoG(PA-LA) would probably affect the absolute peak values for the FPP signal intensity curves. The relative timing, however, of the peak values would not be affected by the pre-bolus.

## Conclusions

The most clinically useful method to assess PBV is with FPP since these data are the same as those acquired during a myocardial perfusion scan and thus comes “for free” without additional contrast or image acquisition. Quantification with CoG(FPP) calculations had lowest inter-observer variability, and best precision with similar accuracy compared to the other FPP methods when taking the LAV into account. Importantly, FPP methods include the LAV in the quantification of PBV and thus provide a summed evaluation of PBV + LAV, a notable distinction since a dilated LA alone can indicate congestion.

## Data Availability

Data will be provided upon request.

## Abbreviations

BSA: Body surface area
bSSFP: Balanced steady-state free precession
CMR: Cardiovascular magnetic resonance
CO: Cardiac output
CoG: Center of gravity
ECG: Electrocardiogram
EDV: End-diastolic volume
EF: Ejection fraction
ESV: End-systolic volume
FPP: First-pass perfusion
ICC: Intraclass correlation
LA: Left atrium/left atrial
LAV: Left atrial volume
LAV_mean_: Mean left atrial volume
LV: Left ventricle/left ventricular
MPA: Main pulmonary artery
PA: Pulmonary artery
PBV: Pulmonary blood volume
PBVI: Pulmonary blood volume indexed to body surface area
PTB: Pulmonary transit beats
PTT: Pulmonary transit time
PV: Pulmonary vein
Q: Flow
ROI: Region of interest
RV: Right ventricle/right ventricular
RVOT: Right ventricular outflow tract
RVSV: Right ventricular stroke volume
SD: Standard deviation
t: Transit time
V: Volume
